# Non-REM parasomnias: a scoping review of dreams and dreamlike mentation

**DOI:** 10.3325/cmj.2022.63.525

**Published:** 2022-12

**Authors:** Omolade Longe, Abiola Omodan, Guy Leschziner, Ivana Rosenzweig

**Affiliations:** 1 Institute of Psychiatry, Psychology and Neuroscience, King’s College London, London, United Kingdom; 2 Department of Human Anatomy, College of Medicine and Health Sciences, University of Rwanda, Kigali, Rwanda; 3 Centre for Neuroimaging, Sleep and Brain Plasticity Centre, IoPPN, King's College London, London, United Kingdom

## Abstract

**Aim:**

To establish patterns or themes of dreams and dreamlike mentation content reported in all forms of non-rapid eye movement (NREM) parasomnias and to identify gaps in the current understanding of this topic.

**Methods:**

A scoping review of available evidence on dreams and dreamlike mentation in NREM parasomnias was conducted in accordance with the PRISMA-ScR guidelines. We searched peer-reviewed literature using Google Scholar, PubMed, Ovid (Embase), Ovid Medline®, Global Health, and APA Psych Info. The Mixed Method Appraisal Tool (MMAT) was used to appraise the quality of selected articles.

**Results:**

The final analysis included 16 studies. All of the studies were from high-income countries. The studies reported on dreams and dreamlike mentation in NREM parasomnias, but there was scarcity of literature for sexsomnia, sleep-related eating disorder, and confusional arousal. All of the studies had the highest quality as shown by the MMAT (76%-100%). Emotions such as apprehension and misfortune were associated with sleepwalking and sleep terrors.

**Conclusion:**

Sleep studies involving collection of dream content immediately following NREM parasomnia could significantly minimize reporting bias and improve dream data quality.

The psychological phenomenon of dreaming has long been most strongly associated with the neurophysiological state of rapid eye movement (REM) sleep (1). Dreaming has been considered a form of consciousness by some researchers, albeit with internally generated perceptions and emotions often found in waking consciousness (1,2). It is often filled with perceptual experiences, predominantly visual, frequently colorful, and usually with well-defined faces of people, animals, objects, and other experiences similar to those of wakefulness (1,3).

Reports on dream content from NREM sleep tend to lack in quality and length (4-10). Attempts have been made to explore and characterize themes associated with dreams in NREM sleep (6). NREM parasomnias, however, offer a natural opportunity to explore the characteristics of NREM dreaming, in that sufferers frequently report some degree of sleep mentation associated with their parasomnia events (1). NREM parasomnias affect people of all age groups, are often marked by skeletal muscle activity or physical actions, and autonomic activation, and sometimes have significant consequences due to injuries and dangers that patients expose themselves and others to (11-13). Patients also frequently suffer from excessive daytime somnolence, pain, and altered quality of life (13).

According to the third edition of the International Classification of Sleep Disorders (ICSD-3) (14), NREM parasomnias (also termed disorders of arousal) include confusional arousals, sleepwalking, sleep terrors, sleep-related eating disorder (SRED), and sexsomnia. More recently, sleep-related choking syndrome has also been considered relevant to this group due to its manifestation during NREM sleep stages (13). Investigators have scrutinized reports of dreams and dreamlike mentation and their contents as a way of gaining a deeper understanding of underlying neurobiology. Most of the investigations have however explored NREM parasomnias within their subtypes. This approach is unlikely to provide a broader view of a set of conditions that have been reported to overlap (15,16). It is therefore reasonable to explore NREM parasomnias as a group. Some priming and precipitating factors for NREM parasomnias have been described (17,18) but these have not been explored more recently. NREM parasomnias are also more prevalent in the psychiatric population, possibly due to the impact of psychiatric disorders and/or psychotropic medications on sleep (15,17,19). Reporting psychiatric disorders associated with NREM parasomnias could further highlight patterns that possibly play a role in developing more effective approaches to managing psychiatric disorders.

In clinical practice, exploration of dream mentation can be used to differentiate REM from NREM phenomena, but the differentiation of REM and NREM dreams (1,20) is no clear. A better understanding of the themes and patterns of dreams and mentation in NREM parasomnias could provide further insight, facilitating the diagnosis of these conditions, and potentially their neurobiological underpinnings (1,13). During our literature search, we found no scoping review looking at the knowledge of dreams and dreamlike mentation in NREM parasomnias. Therefore, we conducted such a review to identify gaps in our current understanding of the topic. The primary research question was “Are there patterns or themes to dreams and mentation in NREM parasomnias reported in adults?” and the secondary question was “If so, can these patterns be defined with sufficient clarity and measured with a good degree of objectivity?”

## Methods

A scoping review of available literature was conducted using the methodological guidelines proposed by Arksey and O’Malley and Levac et al (21-23). Given the overlap between NREM parasomnia types (24-27), data for all NREM parasomnia subtypes were collected.

In determining the eligibility and appropriateness of the primary research question, the framework using Population, Concept and Context was adopted. The outcome of this review was reported using the Preferred Reporting Items for Systematic Reviews and Meta-Analysis (PRISMA) guidelines.

### Data sources and search strategies

In June 2021, we searched six electronic comprehensive databases: Google Scholar, PubMed, Ovid (Embase), Ovid Medline ®, Global Health, and APA Psych Info. Only articles written in English were considered. We used the following search terms: “dreams,” “mentation,” “sleepwalking,” “sleep terrors,” “sleep related eating disorder,” “sexsomnia,” “confusional arousal,” “non-rem parasomnia”, “polysomnography,” and “EEG.” Each database was uniquely searched using Boolean operators “AND” and “OR” in similar sequence. The inclusion criteria were applied all through the screening process. The entire process of the literature search was well documented. All resulting citations were moved into EndNote 20, and the software automatically removed the duplicates. Duplicates subsequently found were removed manually. These citations were then screened using title and abstract contents as well as characterization of the data of the full-text articles.

Studies were eligible for inclusion if they reported qualitative data on dreams or mentation in relation to episodes of NREM parasomnia. Studies comparing dreams and mentation in other parasomnias were also eligible if they included NREM parasomnias and specifically described associated dream and dreamlike mentation. In order to capture as many articles reporting NREM parasomnia dream contents, no restriction was applied to the year of publication. All selected articles included adults (male and female) aged 18 and above referred to sleep clinics/specialists and who had a clinical or polysomnographically confirmed diagnosis of NREM parasomnia, including confusional arousals, sleepwalking, sleep terrors, sleep-related eating disorder, and sexsomnia. For the purpose of this review, episodes of confusional arousals, sleepwalking, sleep terrors, SRED, and sexsomnia were as defined by the third edition of the International Classification of Sleep Disorders (14). We excluded articles reporting on children only, articles reporting dreams and dreamlike mentation in the context of parasomnia overlap disorder (POD), and review articles on dreams and mentation in NREM parasomnias. Agreeability among the co-screeners of the full articles was high (kappa score = 0.989) (28).

### Quality assessment of individual studies

The methodological quality of the selected articles was assessed using the Mixed Methods Appraisal Tool (MMAT) (29) (Supplementary material 1). The articles were subjected to a pilot assessment using a pre-designed form. We assessed how clear each research question was, how the sampling strategy was relevant to the research question, how appropriate the measurements were, and how well the population being studied was represented. Quality was rated as low (≤50%), average (51%-75%), or high (76%-100%).

### Data profile

The dreams and dreamlike mentation described in the case reports were examined in relationship to the reported parasomnia event. Other characteristics of the dream contents reported in all the selected articles were also explored to identify any patterns or themes. To describe and compare the reported dream contents from the selected articles, we used a modified version of the Hall and Van de Castle method of dream content analysis (30). Specific categories were selected to ensure the facts reported in the selected articles remain the same. The dream contents were considered for the presence of characters (people, animals, and objects), social interaction (aggression, friendliness, victim, and sexuality), fortune, misfortune, food/eating, and emotions (anger, apprehension, sadness, confusion, and happiness). The dream contents were characterized as including people when they reported or described presence of human characters other than the dreamer. This is because many of the dream contents are described from the point of view of the dreamer, who is almost always present in the dream experience. Animals were included when there was reference to animals or poorly defined creatures. Objects included inanimate items irrespective of size. Aggression was marked when the contents described deliberately unfriendly behavior either by the dreamer or another character in the dream. Dream contents were marked to include victim when the dreamer suffered an adverse outcome including that resulting from aggression. Sexuality was marked when contents made reference to sexual themes or acts. Fortune was marked for a positive circumstantial outcome, while misfortune was marked for descriptions of mishap, injury, or adversity resulting from an accident, chance, or environmental circumstances. Where food items or the act of eating were mentioned or referred to in dream mentation content, food was marked. All emotions were considered only from the point of view of the dreamer. Anger was marked for content that involved an act of belligerence or expression of anger by the dreamer. Apprehension was considered when contents described a fearful expectation or anticipation; sadness when sorrowful, uncheerful, or mournful events were described; happiness when contentment and cheerful experiences were described, and confusion when unsettling/disorderly feeling, or a sense of perplexity was described.

## Results

**Figure 1:**
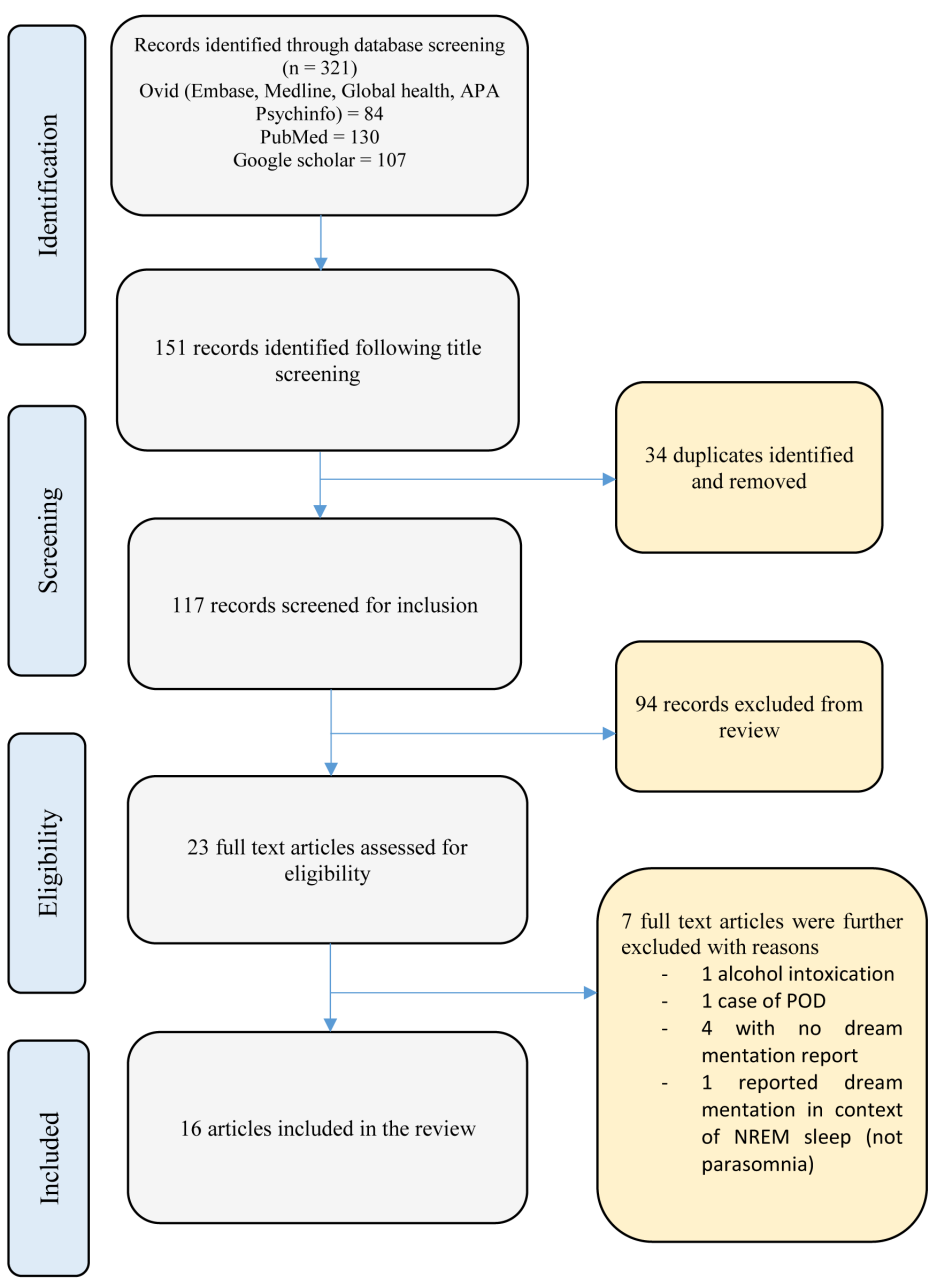
PRISMA flowchart of study selection process. POD – parasomnia overlap disorder; NREM – non rapid eye movement.

The final analysis included 16 articles (Figure 1). Seven articles were excluded during the full-article and data extraction stage: one because the reported dream mentation experience occurred following alcohol intoxication (31); one included a case of POD (32); one reported no recall of dream mentation (33); one was a study of NREM dream mentation but not in the context of NREM parasomnias (34); and the remaining did not describe clear dream mentation (35-37).

### Characteristics of included studies

Of the 16 articles, 10 (62.5%) were case reports (38-47) and 6 (37.5%) were cohort studies (24-27,48,49). All of the studies were conducted in high-income countries. All of the studies used electroencephalogram (EEG)/polysomnography (PSG) as part of diagnostic tools, but not all participants underwent PSG during the reported parasomnia event. The studies enrolled a total of 195 adult participants with NREM parasomnias, with 94 (48.2%) men. All the studies were published between 1991 and 2020. All focused on specific subtypes of NREM parasomnias, although sleepwalking was reported in 10 (62.5%), sleep terrors in 8 (50%), SRED in 3 (18.8%), sexsomnia in 2 (12.5%), and confusional arousals in 1 (6.3%). Of the 195 participants, 27 (13.8%) patients had sexsomnia and 3 (1.5%) patients had confusional arousals (Table 1).

**Table 1:** Characteristics of the included articles

Author(s)	Country of study	Type of study	Non-REM parasomnia described	Number of participants in study with nREM parasomnia	Study aim	Main outcome
Schenck et al (1991)	USA	Cohort study	Sleepwalking Sleep terror SRED	16	To describe polysomnographic correlates of SRED in 19 adults with nocturnal behavior including eating as well as associated sleep-related injury over a 5-year period.	The study concluded sleepwalking, periodic movements of sleep, and triazolam abuse are etiologic categories of sleep-related eating.
Zadra and Nielsen (1998)	Canada	Case report	Sleep terror	1	To describe the topographical EEG mapping study of a patient with sleep terrors, examining one-minute sections of the EEG tracing immediately prior to onset of sleep terror episodes.	The study demonstrated more total and delta power in central and frontal areas compared to control (normative data) and proposed these were possibly related to underlying slow-wave sleep mentation, which may sometimes trigger episodes of sleep terror.
Hartman et al (2001)	UK	Cohort study	Sleepwalking Sleep terror	22	To examine the possibility of underlying protective dissociative process in sleepwalking and night terrors.	They identified 6 patients with a history of trauma and 2 of them demonstrated possible underlying dissociative process with trauma-related sleep mentation content.
Lauerma (2003)	Finland	Case report	Sleep terror	1	To report a case of sleep terror which was proposed to have been related to drug-induced dissociative states in a man following prescription of fencamfamine.	Polysomnography demonstrated evidence of arousals from slow-wave sleep.
Shapiro et al (2003)	Canada	Case reports	Sexsomnia	10	To present case reports describing sexsomnia in 10 adults, some of whom had medico-legal issues resulting from episodes of sleep-related sexual behavior.	The report highlighted similarities as well as distinct differences between sleepwalking and sexsomnia including prominent automatic arousal, restricted but specific motor activities, and associated dream mentation.
Oudiette et al (2009a)	France	Cohort study	Sleepwalking Sleep terror	42	To explore dreamlike mentations during sleepwalking and sleep terrors in adults.	The study concluded complex mental activity takes place during slow-wave sleep, and sleepwalking may represent a form of acting out of mentation.
Pillmann (2009)	Germany	Case report	Sleepwalking	1	To describe a case report of complex dream-enacting behavior in a 26-year-old male patient with sleepwalking	Psychosocial factors and possible worsening of asthma were thought to be contributory factors leading to increased sleep fragmentation.
Brion et al (2012)	France	Cohort study	Sleepwalking sleep terror SRED	36	To compare clinical, sleep and eating behavior measures in patients with SRED against sleepwalkers and controls.	The study concluded the patients with SRED, possibly due to their underlying or pre-existing eating behavior, have somehow specialized their sleepwalking behavior to sleep-related eating behavior.
Perogamvros et al (2012)	Switzerland	Case report	SRED	2	Reported 2 cases of SRED with PSG, psychometric assessment and dream diary.	Study concluded there is possibly an active reward system during sleep, which could be implicated in the manifestation of SRED.
Mwenge et al (2013)	France	Case report	Sleepwalking	1	Reported the case of a 33-year-old woman who monitored her nighttime behavior for 36 days using a home camera.	She reported dreamlike mentation that suggested evidence of dream-enactment. Finger pointing behavior was observed, which demonstrated possibility of visual hallucinations or residual images from dreams projected into the bedroom.
Uguccioni et al (2013)	France	Cohort study	Sleepwalking Sleep terror	32	Compared dream-enacting behaviors in patients with sleepwalking and sleep terrors to dream-enactment in rapid eye movement sleep behavior disorder.	They demonstrated more misfortunes and disasters but less human and animal aggression when compared to RBD group. The differences in the response to threat observed in the dream contents in this study is thought to be one of the many ways dreaming in NREM sleep differs from dreaming in REM sleep.
Szucs et al (2014)	Hungary	Cohort study	Sleepwalking	9	To retrospectively evaluate 13 patients referred to a sleep clinic.	Concluded that, given most cases of sleepwalking are not as extreme as the cases described in their study, violent somnambulism might be a distinct NREM sleep-related overlap parasomnia.
Dubessy et al (2017)	France	Cohort study	Sleepwalking Sexsomnia	17	Described patients with sexsomnia and compared clinic and sleep measures with those of sleepwalkers and controls.	The authors concluded there was male predominance of sexsomnias and pointed out possibility of a continuum with sleepwalking on one end and sexsomnia on the other.
Iqbal et al (2017)	USA	Case report	Sleep terror	1	Described a 21-year-old female with history of anxiety and depression.	This case highlighted the unusual presentation of what the authors concluded were hypnopompic hallucinations in stage 2 of NREM sleep.
Gnoni et al (2020)	UK	Case report	Confusional arousal	3	Described 3 rare cases of nihilistic delusions (*Cotard le délire de negation*) in which affected individuals described complete or partial loss of their perception of body and existence	Proposed Cotard parasomnia and its associated phenomenology may be the product of incomplete, fluctuating activation and switching between the salience network and the default mode network.
Rocha and Arnulf (2020)	France	Case report	Sleepwalking Sleep terror	1	Reported a case of dream-enactment in a 37-year-old woman with a history of sleep terrors and sleepwalking.	The unique links of the dream content with reality and the recall of events led the authors to conclude NREM parasomnias can also be dream-enacting behaviors.

*Abbreviations: SRED – sleep-related eating disorder; PSG – polysomnography; NREM – non rapid eye movement; RBD – REM sleep behavior disorder.

### Quality of evidence from included studies

All of the 16 qualified studies had MMAT scores in the highest range (76%-100%).

### Dream mentation themes from included studies

**Figure 2:**
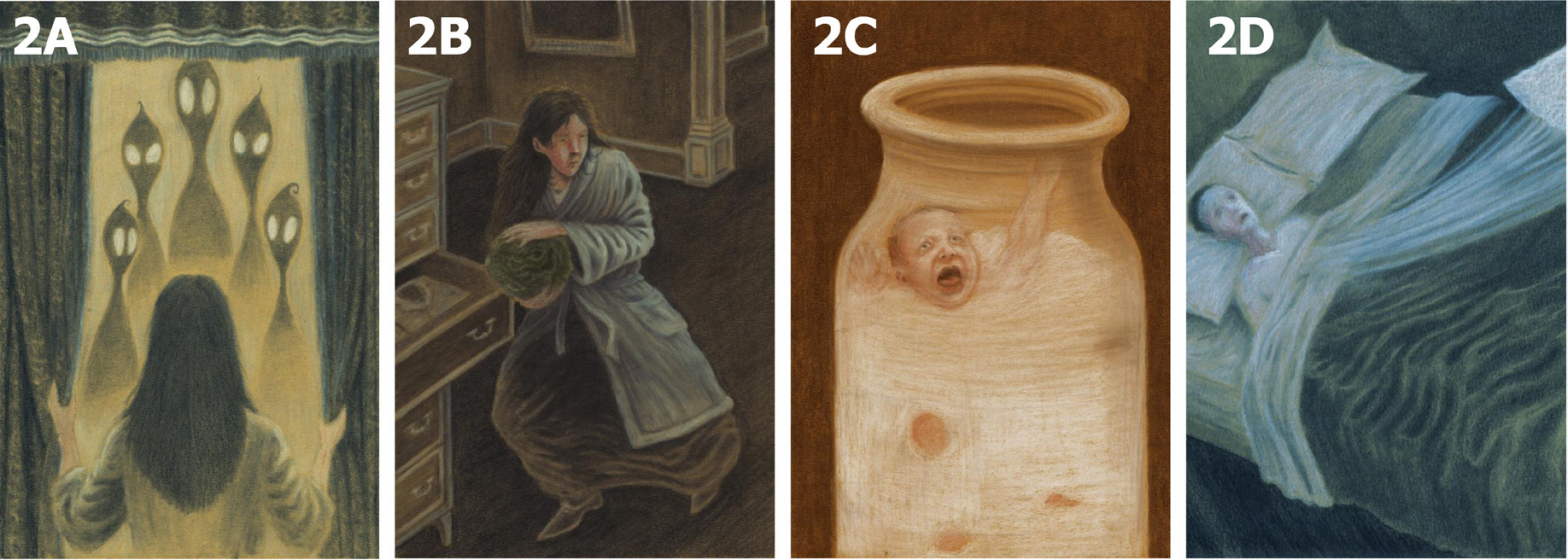
Illustrations (by Davor Aslanovski) of selected dreamlike mentations reported in the studies in Table 2. A) “Pulling down curtains while dreaming of creatures staring at her” (27); B) “Dreaming of finding a safe place for lettuce” (27); C) “Child drowned in a jar of milk” (41); D) “I am dead. I do not exist. I worry if I will be able to return into my body and I have to pinch myself to check if my body is alive” (47).

All of the included articles reported at least one dream mentation content, however seven characterized the dream content in relation to the NREM parasomnia observed (26,40,41,43,47-49). A total of 115 patients reported dreamlike mentation. There was a preponderance of men in the studies on sexsomnia (20 men vs 7 women) (25,45) and a preponderance of women in the studies on SRED (28 women vs 10 men) (24,27,46). Table 2 presents examples of dreams and dreamlike mentation content as described in some of the included studies. Figure 2 illustrates some of the dreamlike mentation contents described.

**Table 2:** Representative examples of dreamlike mentation description reported in included studies

Study	Dreamlike mentation content as reported/described	NREM Parasomnia types reported in study
Oudiette et al (2009a)	Plunged to death Locked in a box and could not escape. Feeling suffocated Broke vial containing a lethal virus Being threatened by a Chinese dragon	Sleepwalking/sleep terrors
Uguccioni et al (2013)	People trying to kidnap my sons so I attacked them. I could not see the face of the aggressors A truck drove straight at me so I tried to avoid it and run away I was walking on the street and saw my husband kissing another girl so I went to him but a girl was trying to stop me. She pulled on my arm so I gave her a slap	Sleepwalking/sleep terrors
Szucs et al (2014)	Dangerously confined in a small place and unable to leave Endangered due to flooding and needed to escape Feeling trapped in a narrow tunnel with a train fast approaching	Sleepwalking
Mwenge et al (2013)	Finding baby trapped under the sheets, furniture and under husband's body Child drowned in a jar of milk Ceiling collapsing	Sleepwalking
Gnoni et al (2020)	My body is drained of blood and I know I am dying or dead I am dead. I do not exist; I worry if I will be able to return into my body and I have to pinch myself to check if my body is alive I am rising above my body, my body feels empty, and my soul has left my body as a large lump of sugar	Confusional arousal
Zadra and Nielson. (1998)	There was a man sitting in a chair beside her bed who wanted to harm her Described “her mouth had been filled with large insects which she was quickly trying to grab with her hands and spit out – but without success” Vague recall about “choking on something”	Sleep terror
Schenck et al (1991)	Finding a safe place for lettuce Guests arriving for dinner party (while having episode of SRED) Pulling down curtains while dreaming of creatures staring at her	SRED/Sleepwalking/Sleep terror
Hartmann et al (2001)	Trying to flee from an attacker Attempting to fight a male attacker Seeing snakes in bedroom and feeling of being pursued by an evil presence	Sleepwalking/Sleep terror

*Abbreviations: SRED – sleep-related eating disorder; NREM – non rapid eye movement.

### Subtypes of NREM parasomnia

Ten articles (62.5%) included patients with sleepwalking (24-27,40,41,43,44,48,49), 9 (56.3%) included patients with sleep terror (24,26,27,38,39,42-44,48), 2 (12.5%) included patients with sexsomnia (25,45), 3 (18.8%) included patients with SRED (24,27,46), and 1 study (6.25%) included patients with confusional arousals

### Presence of characters

In 10 (62.5%) articles, dream contents involved people other than the dreamer (26,27,38-41,44,45,48,49). None of the articles involved patients with confusional arousals. Seven (43.75%) articles (24,26,38,41,42,44,49) reported dreamlike mentation content involving animals or bizarre creatures. Patients with sexsomnia or confusional arousals reported no animals in their dream content.

### Social interaction, dream content experience, and emotions evoked

Most of the patients described one scene, often lacking context and detail. The scene often involved the dreamer experiencing or witnessing an event. One of the themes identified was aggression. This included intentional and unfriendly behavior exhibited by a character (dreamer, animal or others). Six articles (37.5%) reported dream content consistent with the above description of aggression (26,38,39,44,48,49). None of the articles describing aggressive content involved patients with SRED, sexsomnia, or confusional arousals.

The experience of dream content from the perspective of the dreamer can also be viewed as a positive or negative one. Reported dream content from each of the included articles was characterized as either fortune or misfortune. Reported dreamlike mentation content involving misfortune was noted in 9 (56.25%) articles (24,26,38,41-43,47-49). In articles on sexsomnia, dream content involving misfortune was not reported. They were however the only articles reporting dream content interpreted as fortune. The prevailing emotional theme associated with dream content was apprehension. It was reported in all but 2 (12.5%) articles (25,45), both of which involved patients with sexsomnia. In sleepwalkers and sleep terror patients, Oudiette et al (26) found apprehension in 84% (89 of 106) of dreamlike mentation contents, while Uguccioni et al (48) described threatening events in 92% (68 of 74) dreamlike mentation contents.

### Dream enactment associated with NREM dreamlike mentation

Eight (50%) articles reported enactment of dreamlike mentation (26,27,38,40,41,43,48,49). Two of the case reports (40,43) each described unique cases of NREM dream enactment that seemed to incorporate episodic memory of real-life events within dreamlike mentation content, supporting the evidence of isomorphism of dreamlike mentation and associated parasomnia event as well as the possible influence of psychosocial stressors in dream enactment. Cases of violent sleepwalking appeared to be highly associated with enactment of dreamlike mentation (49). One of the included studies (48) found that over 90% of sleepwalkers and sleep terror patients reported enactment of dream content. Dream mentation content was collected during PSG (immediately after parasomnia event) in only 3 of the studies (38,43,48), which makes reports of dream content less reliable.

### Precipitating factors and psychiatric data

Precipitating factors for episodes of arousal and associated dream content enactment included alcohol intake, sleep deprivation, stress or argument, and psychological trauma (24,26,44). The precipitating factors for sleepwalking episodes with enactment of dreamlike mentation content were movement of bed partner or pet, or pre-existing physical health problems such as asthma (40,41), and those for episodes of confusional arousals were other sleep disorders including periodic limb movements and obstructive sleep apnea (47).

Patients with NREM parasomnia were frequently reported in to have psychiatric conditions. The reported conditions included anxiety, affective disorders, posttraumatic stress disorder, depression, bipolar affective disorder, mild cognitive impairment, intellectual disability, schizophrenia, and conversion disorder (25,39,44,45,47,49).

## Discussion

Our findings highlight a dearth of literature reporting dreams and dreamlike mentation in patients with confusional arousal, sexsomnia, and SRED. However, they also indicate commonalities in dream content across some, if not all, parasomnia types (1). Certainly, our analysis demonstrates an abundance of literature describing mental activity involving characters, particularly in patients with sleepwalking and sleep terrors. A lack of available literature exploring this was observed in patients with sexsomnia, confusional arousals, and to some degree, SRED. The paucity of evidence related to confusional arousals may, at least in part, be explained by lack of full awakening after these types of events. Emotional themes including aggression, misfortune, and apprehension are frequently experienced across NREM parasomnia types, although there is little evidence to support these themes in sexsomnia. In fact, sexsomnia appears to be an outlier in terms of dream content, a finding that perhaps implies a subtly different underlying pathophysiology. The widespread view of NREM parasomnias as automatic and amnesiac behaviors has been recently increasingly challenged (13). Instead, subjective and objective observations suggest that sleepwalkers are “dream-walking” during their episodes due to local arousals and a wake-like activation in motor and limbic regions and a preserved, sometimes increased, inhibition of the frontoparietal network.

In keeping with this, our findings also support the view that NREM parasomnias are linked to stress, arguments, and psychological trauma, and that enactment of dream content may be related to both violence and real-life events. This suggests that enactment may be driven or triggered by the emotional experience of dreams and dreamlike mentation or by stress, leading to increased likelihood of localized arousals during sleep, as well as to possible hyperactivity of the amygdala and emotional network (26).

Few of the selected articles reported precipitating factors for the investigated NREM parasomnia types. However, the frequency of precipitating factors was not reported. A wide range of psychiatric conditions was associated with NREM parasomnias, but no clear pattern was identified with regards to particular NREM parasomnia types.

Our study was largely limited by the primary lack of dream contents or poorly reported dream contents in the articles considered. This highlights the insufficiency of research publications describing dreams and dreamlike mentation content in NREM parasomnias. Moreover, all 16 articles were written in high-income countries, demonstrating a lack of studies reporting dreams and dreamlike mentation content in middle-income and low-income countries. This study was further limited by selection criteria requiring only studies that validated NREM parasomnias via PSG and studies reported in the English language. Another limitation relates to the timing of dream content collection, which for the most part was performed retrospectively, or only after certain time has passed. This may be the reason for the general lack of detail, perhaps due to poor recall or confabulatory elements (50). In just three of the included studies, dream content was collected immediately following PSG evidence of NREM parasomnia, which potentially casts doubt on the reliability of the dream contents described.

Further analysis of dream content associated with NREM parasomnia subtypes may be helpful in understanding differences in the neurobiological basis of these different phenotypes, but ideally should be performed through the proactive collection of dream content during PSG wherever possible (1,13,50). In clinical practice, this may be challenging, as complex behaviors are only seen in approximately 30%-35% of PSGs undertaken in patients with a NREM parasomnia (1).

Finally, the dream content associated with NREM parasomnias may not be representative of that in normal NREM sleep. Dream analysis in normal NREM sleep has been performed through serial awakening paradigms (51), but such studies remain limited. Differences between dreams in NREM parasomnias and normal NREM sleep may provide important clues as to the nature of these disorders.

In conclusion, dreams and dreamlike mentation content in NREM parasomnias are generally poorly documented, particularly in less common forms, such as SRED and sexsomnia. However, these findings imply that there may be some important differences in dream content between sub-types of NREM parasomnias and their external manifestations. These should be systematically studied as they may provide relevant clues to the underlying neurobiological drivers.
